# Supplementary material for: Insights into the mechanism of action of the arbitrium communication system in SPbeta phages
Source: Nat Commun. 2022 Jun 24;13:3627. doi: 10.1038/s41467-022-31144-3 (PMC9232636; doi:10.1038/s41467-022-31144-3)
Supplement: Supplementary file 1 — Supplementary information [file 41467_2022_31144_MOESM1_ESM.pdf]

Supplementary Information

Insights into the mechanism of action of the arbitrium communication system present in SPbeta phages

Francisca Gallego del Sol[1], Nuria Quiles-Puchalt[2,3], Aisling Brady[2,3], José R. Penadés[2,3*], Alberto Marina[1*].

[1]Instituto de Biomedicina de Valencia (IBV-CSIC) and CIBER de Enfermedades Raras (CIBERER), C/ Jaume Roig 11, 46010, Valencia, Spain

[2]Institute of Infection, Immunity and Inflammation, College of Medical, Veterinary and Life Sciences, University of Glasgow, Glasgow, G12 8TA, UK

[3]MRC Centre for Molecular Bacteriology and Infection, Imperial College London, SW7 2AZ, UK.

*Correspondence: amarina@ibv.csic.es (A.M.)
JoseR.Penades@glasgow.ac.uk (J.R.P.)

CONTENTS

Supplementary Figures

	page
Supplementary Figure 1	3
Supplementary Figure 2	4
Supplementary Figure 3	5
Supplementary Figure 4	6
Supplementary Figure 5	7
Supplementary Figure 6	8
Supplementary Figure 7	9
Supplementary Figure 8	10
Supplementary Figure 9	11
Supplementary Figure 10	12

Supplementary Tables

Supplementary Table 1	13
Supplementary Table 2	14
Supplementary Table 3	15

References 16

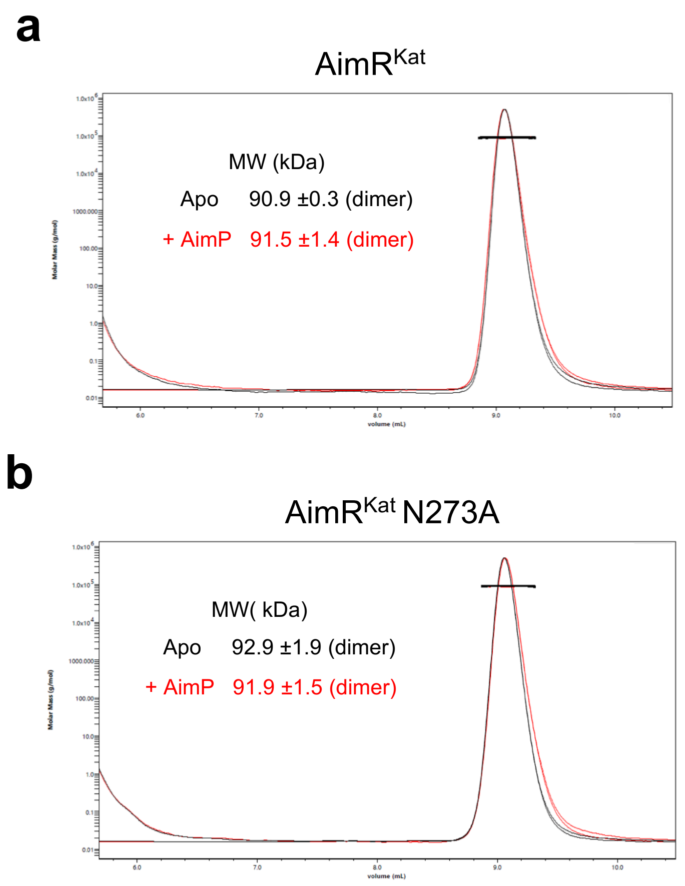

Supplementary Fig. 1. AimR^{kat} is a dimeric protein. Size exclusion chromatography-multi-angle light scattering (SEC-MALS) chromatograms of **a** AimR^{kat} and **b** AimR^{kat}-N273A in absence (black) and presence (red) of AimP^{kat} peptide (GIVRGA). Chromatograms show the readings from the light scattering (dashed line) and refractive index (continuous line) detectors. The vertical axis represents the molecular mass. The horizontal curves represent the calculated molecular masses. In all cases the molecular weight calculated corresponds to a dimer (90kDa).

		APO	Peptide	Crystallization Conditions
AimR^{Kat}		**Closed** 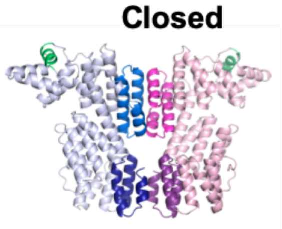 *PDB:* 6S7I *Group*: P2$_1$2$_1$2$_1$ *Cell* (Å) a=77,6 b=98.3 c=144.4 *Tag:* Removed	**Closed** 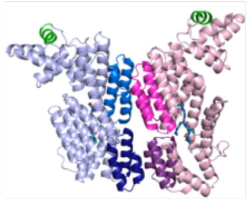 6S7L C222$_1$ a=69.7 b=209.8 c=140.3 Removed	**Apo**: , 0.1M HEPES (pH 7.0), 0.1M $LiSO_4$, 30 % w/v Polyvinylpyrrolidone **Peptide:** 0.1M MES (pH 6.0),0.2M Potassium acetate, 15% Pentaerythritol ethoxy, 3% Jeffamine T-403
AimR$^{SP\beta}$	AimR$^{SP\beta}$-I	**Closed** 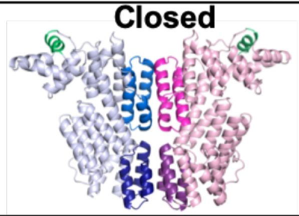 6HP3 P2$_1$ a=78.9 b=251,2 c=95,1 b=90.9 Removed	**Closed** 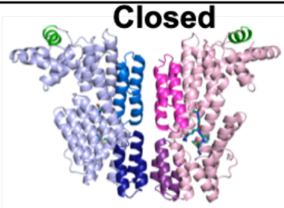 6PH5 P2$_1$ a=54.1 b=79,1 c=55.2 b=106.6 Removed	**6HP3**: 0.1 M MES (pH 6.5), 0.55 M NaCl, 15%PEG4000. **6HP5**: 1.5 M $(NH_4)_2SO_4$
	AimR$^{SP\beta}$-II	**Open** 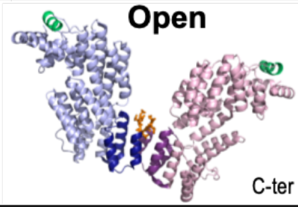 6JG5 P2$_1$2$_1$2 a=115,3 b=219.4 c=33.5 C-ter LEHHHHHH	**Open** 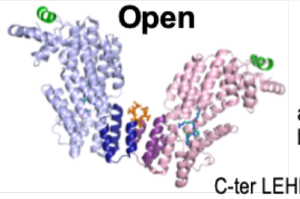 6JG9 P2$_1$2$_1$2 a=120,9 b=214.0 c=33.6 C-ter LEHHHHHH	**6JG5**: 0.1 M Na cacodylate (pH 6.6), 11% PEG 8000, 0.2 M NaCl **6JG9:** 0.1 M Na cacodylate (pH 6.1), 9% PEG 8000, 0.2 M NaBr,, 5 mM DTT
		**Open** 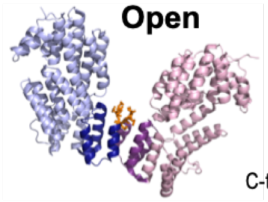 5XYB P2$_1$2$_1$2 a=114,8 b=220.4 c=33.6 C-ter LEHHHHHH	**Open** 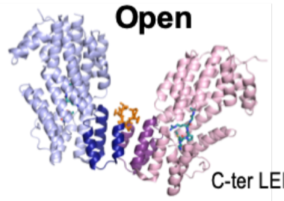 5Y24 P2$_1$2$_1$2 a=119,6 b=214.4 c=33.6 C-ter LEHHHHHH	**5XYB:** 0.1 M Na cacodylate (pH 6.0) 10% PEG 8000, 0.2 M NaCl **5Y24:** 0.1 M Na cacodylate (pH 6.1), 9% PEG 8000, 0.2 M NaBr, 5 mM DTT
		**Open** 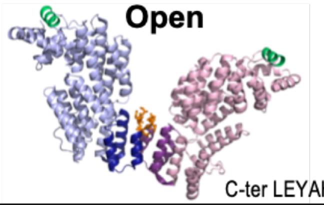 5ZW5 P2$_1$2$_1$2$_1$ a=124,7 b=207.1 c=33.3 C-ter LEYAHHHHHH	**Open** 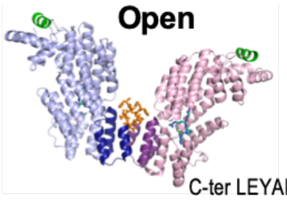 5ZW6 P2$_1$2$_1$2 a=150,8 b=193.6 c=33.2 C-ter LEYAHHHHHH	**5ZW5:** 200 mM $CaCl_2$, 20% PEG3350 **5ZW6:** 100 mM sodium-citrate (pH 4.0), 200 mM citric acid, 20% PEG3350
		**Open** 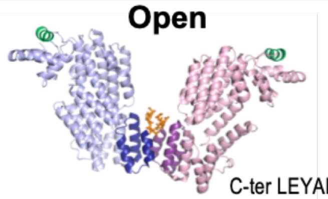 6IPX P2$_1$2$_1$2 a=120,5 b=214.7 c=33.7 C-ter LEYAHHHHHH	**Open** 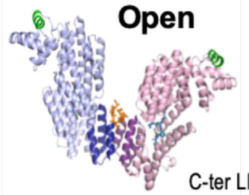 6IM4 P2$_1$2$_1$2 a=121,7 b=214.2 c=33.9 C-ter LEYAHHHHHH	**6IPX:** 0.1 M HEPES (pH 6.5-7.5), 20% (v/v) PEG 4000, 50 mM magnesium acetate **6IM4:** 0.1 M HEPES (pH 6.5-7.5), 20% (v/v) PEG 4000, 50 mM magnesium acetate
AimR^{Phi}		**Closed** 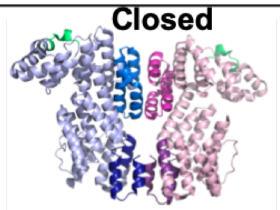 5ZVV P2$_1$ a=61.5 b=116,9 c=68,2 b=113.6 Removed	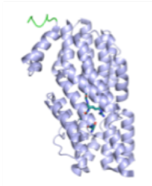 5ZVW P32 a=59,3 b=59.3 c=104,7 Removed	**5ZVV:**100 mM Bis-Tris (pH 6.5–7.0), 2% Tacsimate (pH 6.0), 20% PEG3350 **5ZVW:**, 100 mM Bis-Tris (pH 5.5), 200 mM $(NH_4)_2SO_4$. 20% PEG3350

Supplementary Fig. 2. AimR reported structures. The structures for AimR^{kat,} AimR$^{SP\beta}$ and AimR^{Phi} in their apo and AimP-bound are shown in cartoon rendering with protomers colored in blue and pink. Dimerization Interfaces are highlighted with brighter or darkest tones the slipping surface or capping helices, respectively. DNA recognition helices $\alpha 3$ are highlighted in green. For each structure its PDB code, space group, cell size and the presence of tags in the crystallized protein as well as the crystallization conditions are indicated. Depending on the use of one or two dimerization surfaces, the structures are classified as presenting open or closed conformation, respectively.

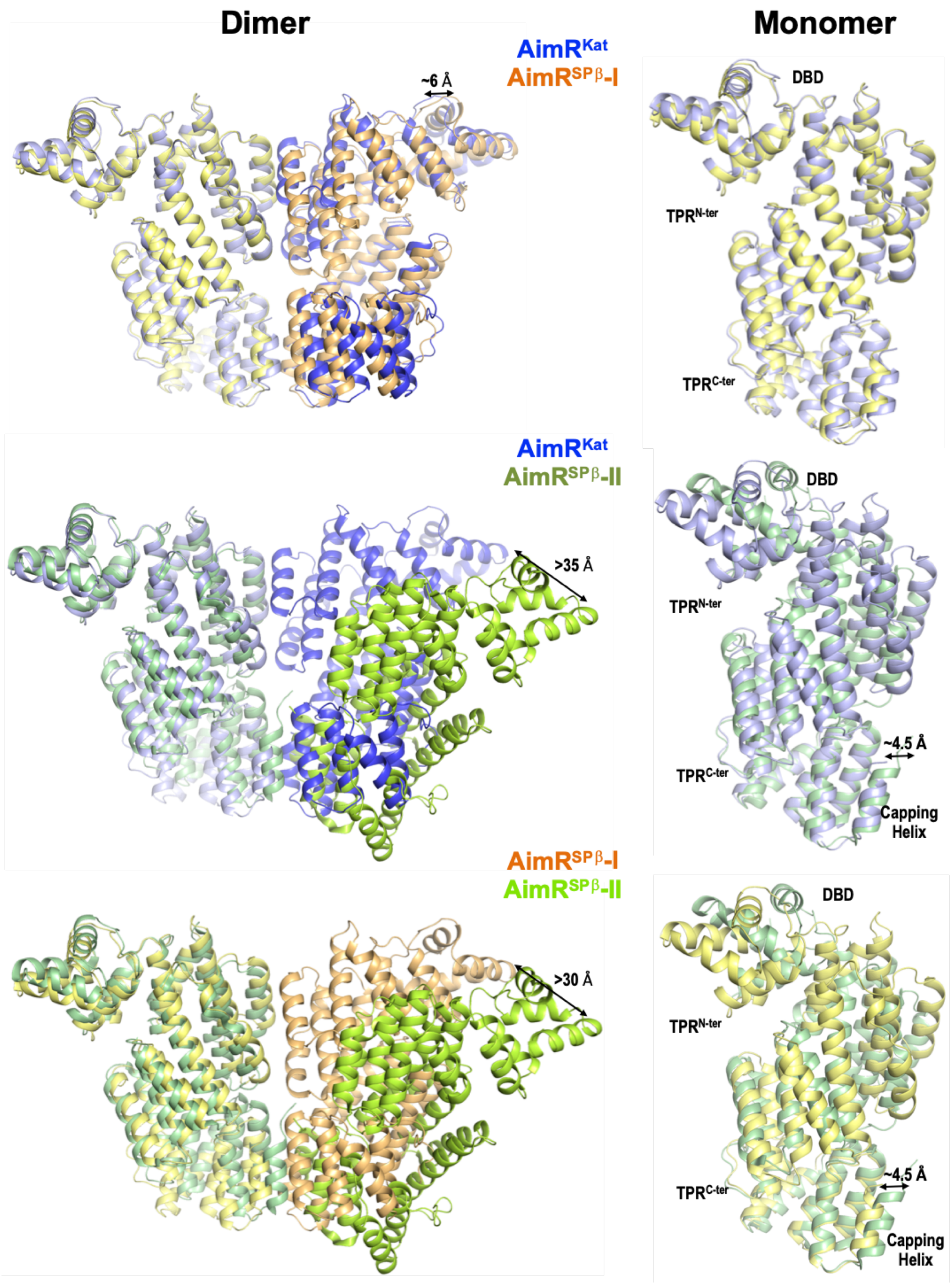

Supplementary Fig. 3. Comparison of AimR receptors in the Apo state. On the *left* is shown the pairwise comparison of $AimR^{kat}$ (blue tones), $AimR^{SP\beta}$-I (yellow-orange shades; PDB 6HP3) and $AimR^{SP\beta}$-II (green shades; PDB 6IPX) receptors in apo state by superimposing a protomer (left) of these dimers. While the second protomer is packed closed for the $AimR^{kat}$ and $AimR^{SP\beta}$-I dimers, showing both a very similar dimeric organization, in $AimR^{SP\beta}$-II the second protmer moves away (more than 30 Å with respect to the apposition of the second protomer in the other structures) showing an open conformation. Superimposition of the individual protomers (*right*) shows an almost identical conformation in all the structures with only small displacements in the capping helices used by AimRs to dimerize that, in the case of $AimR^{SP\beta}$-II, includes the extra His-tag.

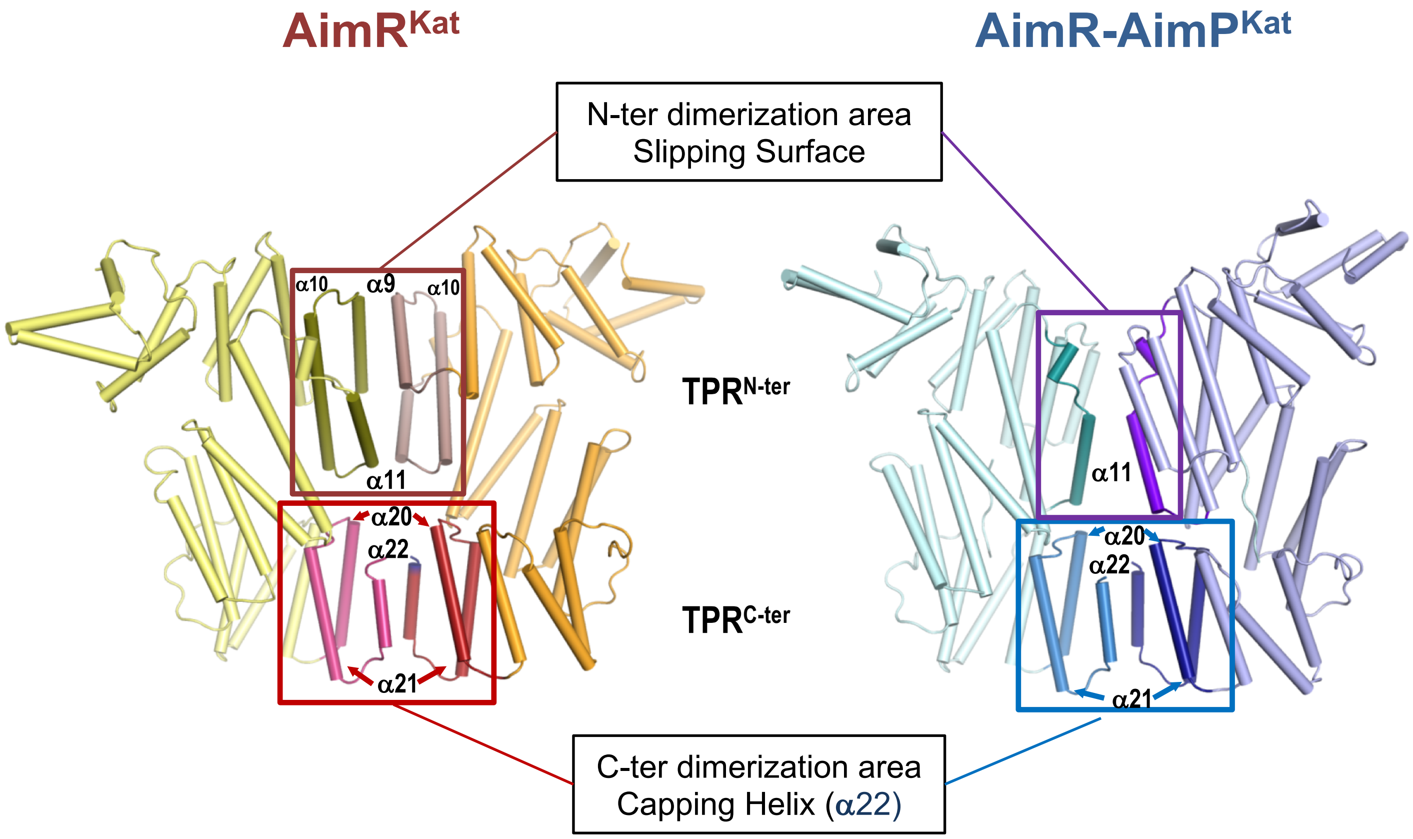

Supplementary Fig. 4. Two dimerization surfaces allow AimP-induced rearrangements to AimR receptors. Cartoon rendering with helices as cylinders of AimR^{kat} dimers in apo (left; in yellow-orange tones) and AimP-bound (*right*, blue tones) states. The two dimerization areas presented in the dimer are highlighted with more intense tones and the structural elements participating in the interactions are labelled. While the C-terminal dimerization area, including the capping-helix, maintains identical contacts in both structures, the N-terminal area acts as a slipping surface changing the interactions between structures.

a

	No peptide	AimP^{Kat} GIVRGA	AimP$^{SP\beta}$ GMPRGA	AimP^{Phi} SAIRGA
Tm (ºC)	41	61	43	43

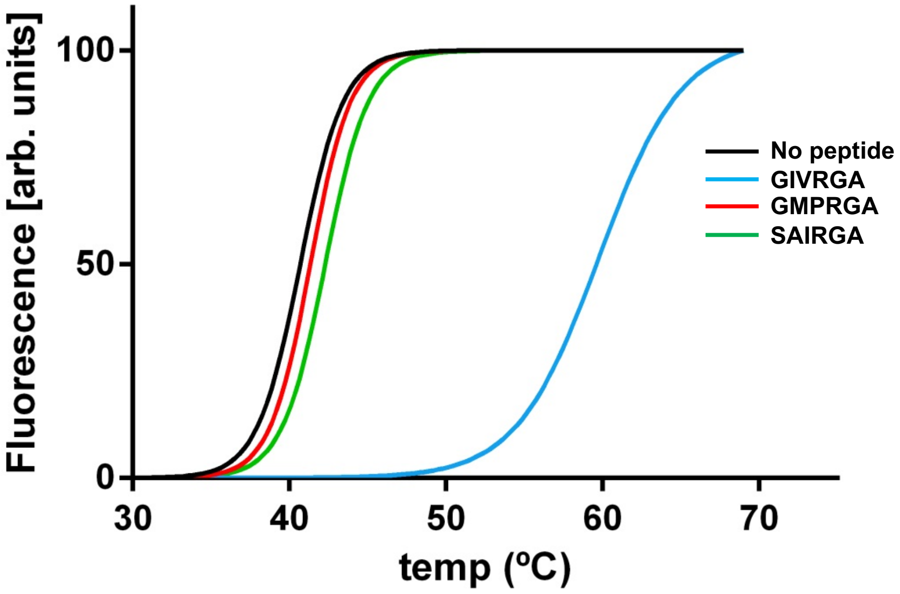

b

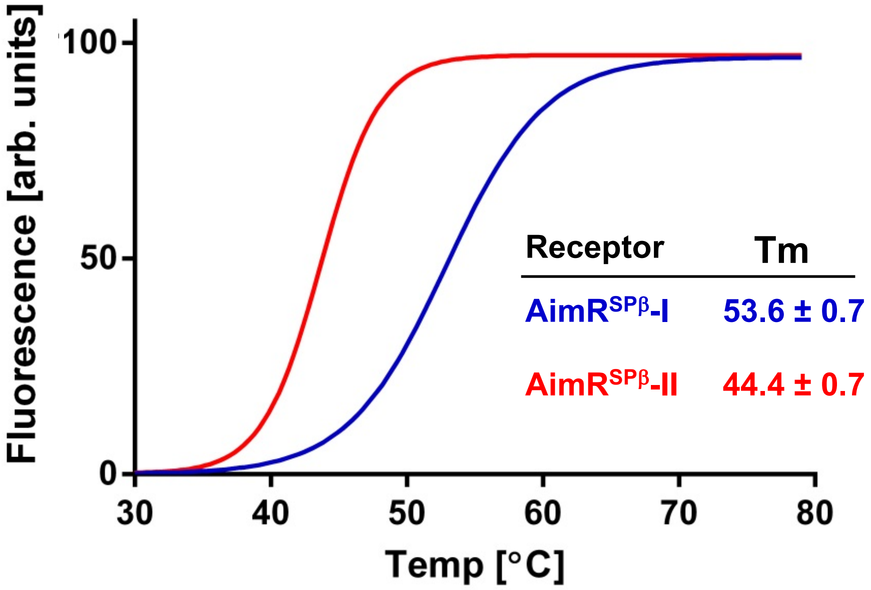

Figure 5. Thermal shift assay for AimR^{Kat} and AimR$^{SP\beta}$. a The denaturation Tm of AimR^{Kat} in its apo form (black line) shows and increment in the presence of GIVRGA peptide (blue line), its AimP, but not in the presence of GMPRGA peptide (red line) or SAIRGA peptide (green line), the AimPs from SPβ and Phi3T phages, respectively. All peptides were assayed at 0.5mM concentration. **b** AimR$^{SP\beta}$ stability decreases by the presence of C-terminal His-tag how confirms denaturation curves of AimR$^{SP\beta}$ with (AimR$^{SP\beta}$-II; red curve) and without (AimR$^{SP\beta}$-I; blue curve) this tag. Source data are provided as a Source Data file.

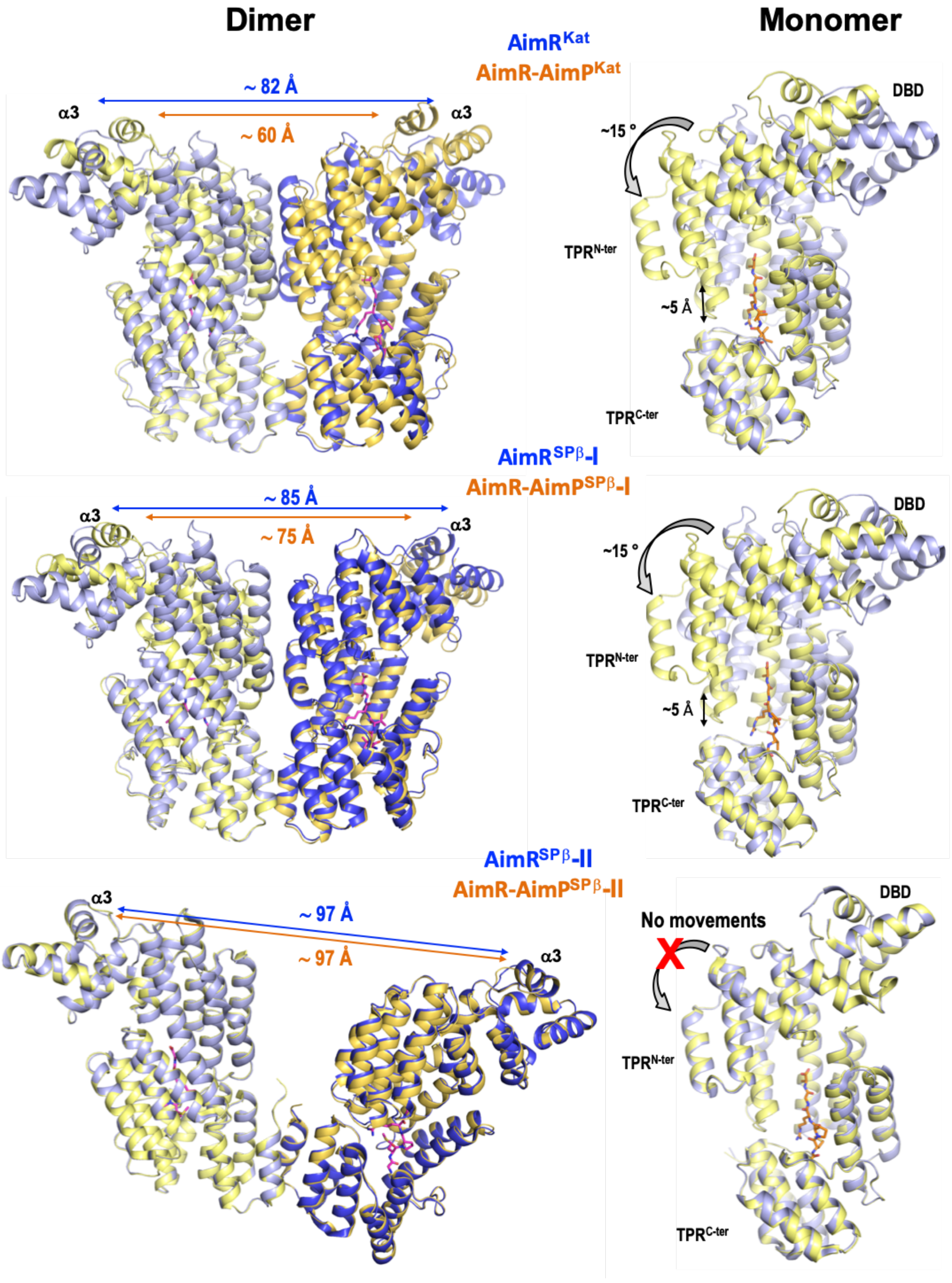

Supplementary Fig. 6. AimP-induced conformational changes in AimR receptors. Structural comparison of AimR^{kat}, AimR^{SPβ}-I and AimR^{SPβ}-II receptors in their apo (blue tones) and AimP-bound (yellow-orange tones) states. The structural superimposition shows how AimP binding induces in the protomer (*right*) a closure movement that brings together TPR^{N-ter} and TPR^{C-ter} domains for the AimR^{kat} and AimR^{SPβ}-I structures. These conformational changes translate to the dimers (left) in the reduction of the distance between α3 helices. On de contrary, AimP does not induce any changes for AimR^{SPβ}-II whose protomers present identical conformations in both states and, consequently, the corresponding dimers are structurally identical.

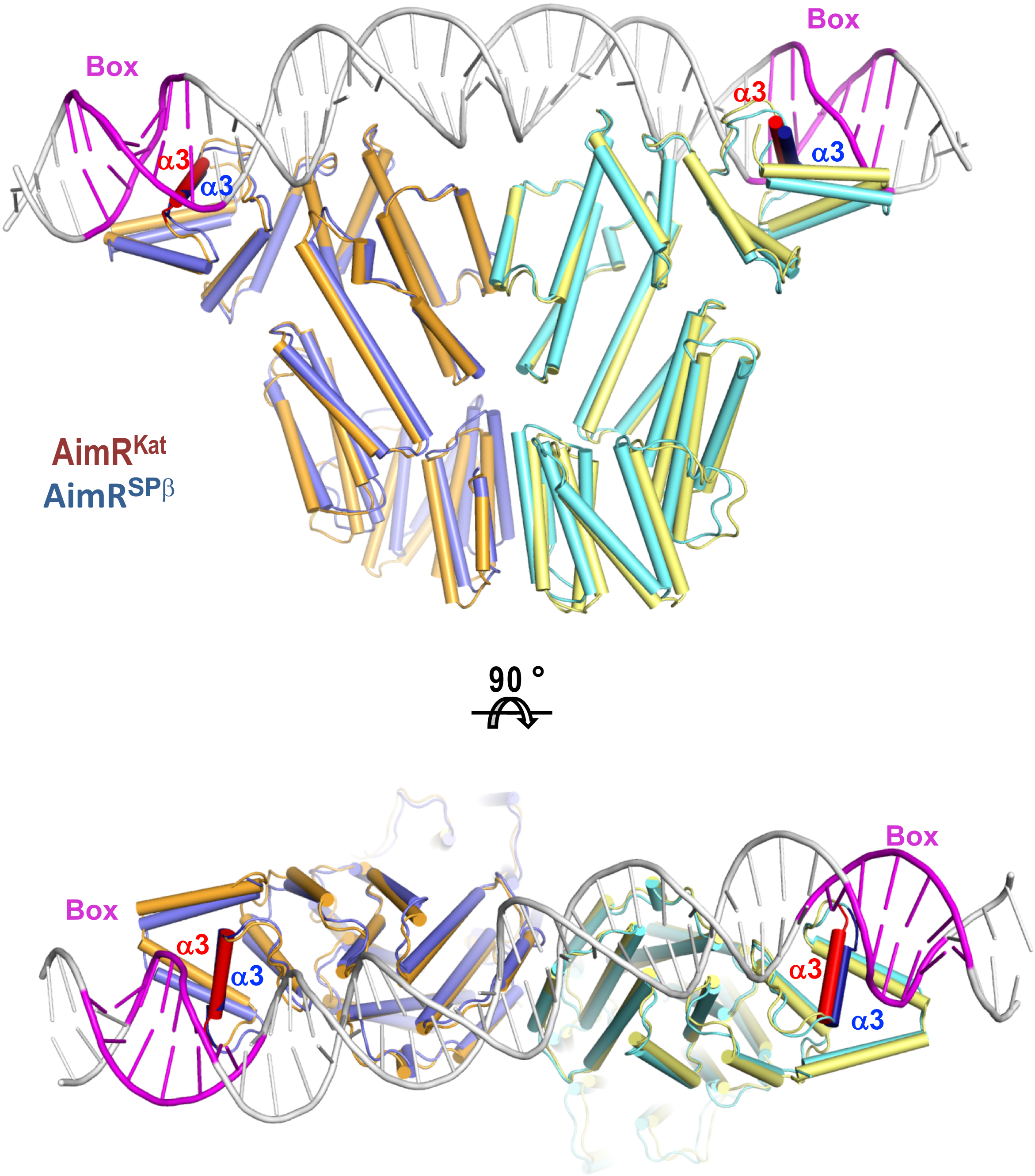

Supplementary Fig. 7. AimR^{kat} in its apo state presents a DNA-binding competent conformation. The superposition of AimR^{kat} in its apo stated (yellow-orange tones) on the DNA-bound AimR$^{SP\beta}$ structure (PDB 6pH7; blue-cyan tones) shows that the DBD domains and the α3 helices (darker tones) occupy identical positions in both structures and, therefore, the helices are prefect positioned for the DNA boxes (highlighted in magenta) read-out. Two orthogonal views are shown with the AimRs rendered in cartoon and the DNA in backbone.

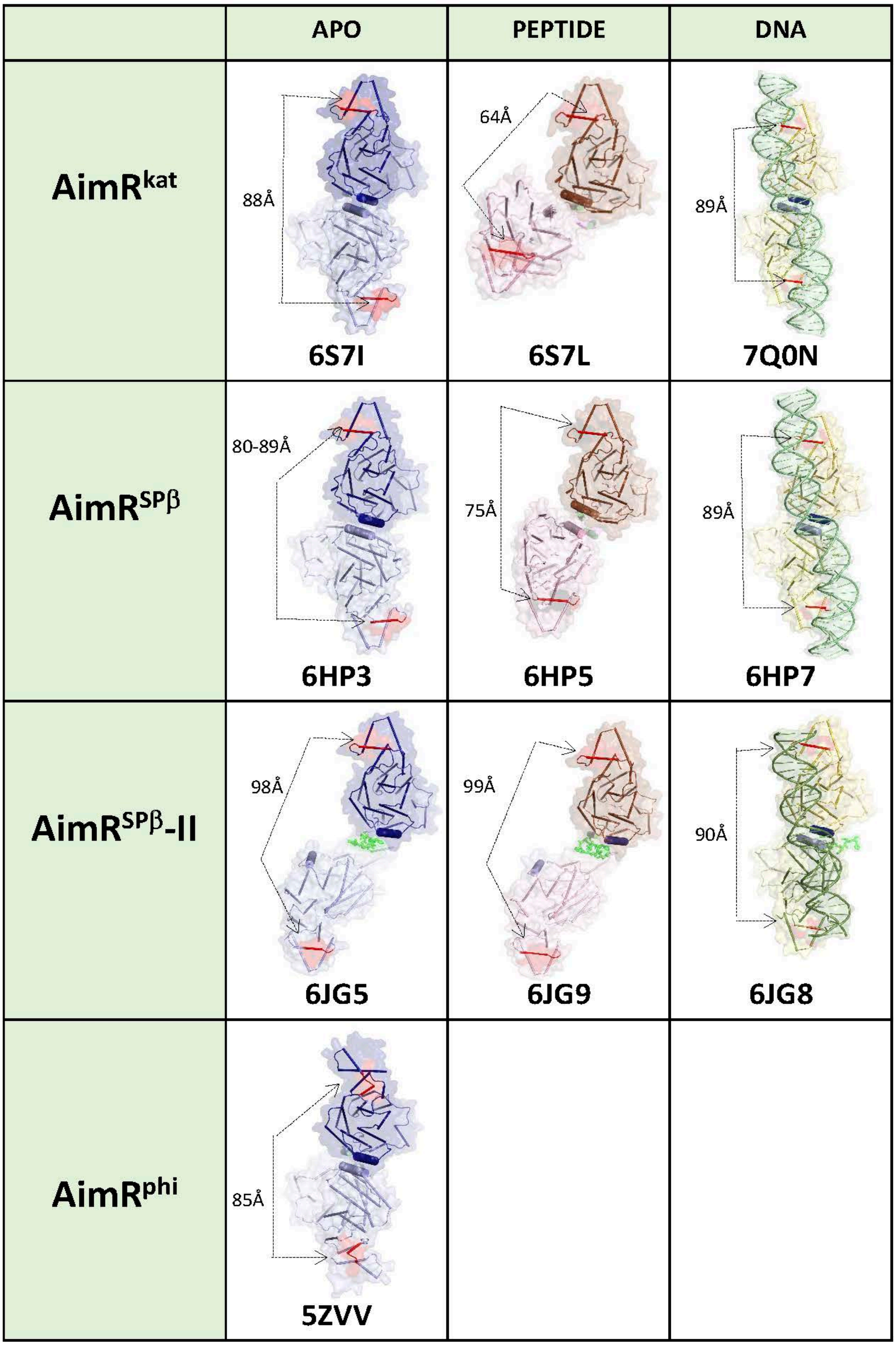

Supplementary Fig. 8. Distances between DNA binding helices in AimR reported structures. The structures for AimR^{kat,} AimR$^{SP\beta}$ and AimR^{Phi} in their apo (colored in blue tones), AimP-bound (colored in pink tones) and DNA-bound (colored in yellow tones with DNA in green) are shown in cartoon rendering, with protomer A in darker tones. Dimerization slipping Interfaces are shown with cylindrical helices. DNA recognition helices α3 are highlighted in red, with distances between α3 helices of protomers A and B indicated in each case. C-terminal His-tag C are shown in sticks and coloured in light green in those structures where they are present. For each structure its PDB code is indicated.

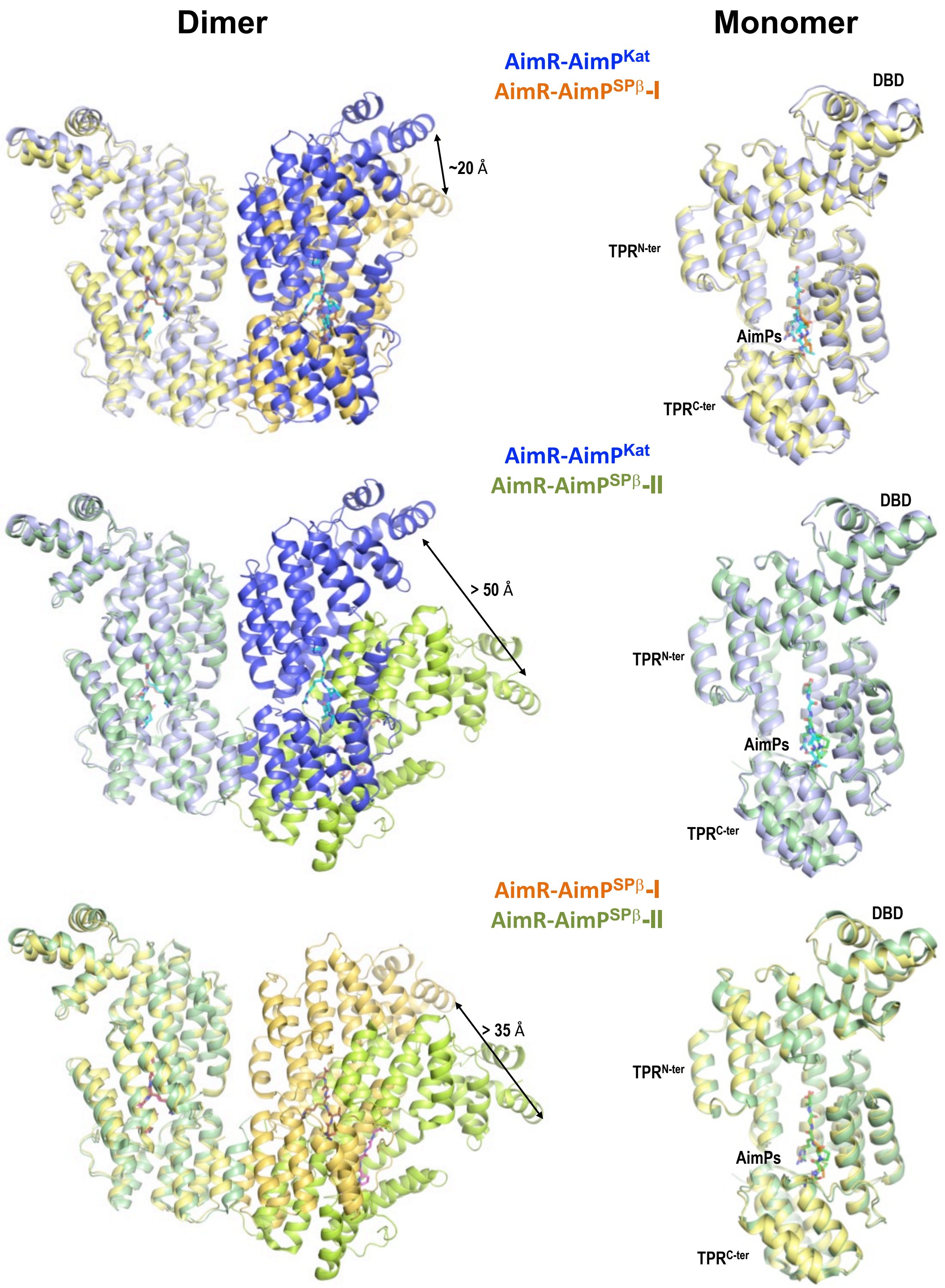

Supplementary Fig. 9. Structural comparison of AimR receptors in their AimP-bound state. The superposition of AimR^{kat}, AimR$^{SP\beta}$-I and AimR$^{SP\beta}$-II receptors bound to their cognate AimPs shows that the individual protomers (*right*) present identical conformation. However, the organization of the dimers (*left)* is quite different, changing the relative disposition of the second protomer, which is surprising in the comparison of AimR$^{SP\beta}$-I with AimR$^{SP\beta}$-II since both structures correspond to the same protein in complex with the same peptide.

a

Name	Strain	Genome ID	AimR NCBI Ref	AimP NCBI Ref
SPbeta	*Bacillus subtilis*	NC_001884.1	WP_009968986.1	WP_009967508.1
Katmira	*Bacillus subtilis*	JMEF01000083.1	WP_033885437.1	WP_134819006.1
SRCM102756	*Bacillus subtilis*	NZ_CP028218.1	WP_160244980.1	WP_160243822.1
NMTD54	*Bacillus atrophaeus*	NZ_PVQN01000007.1	WP_106034888.1	WP_142394717.1
Phi3T	*Bacillus subtilis*	KY030782.1	WP_153256842.1	WP_134982144
JNUCC	*Bacillus subtilis*	NZ_VPFB0.000001	WP_147772092.1	WP_147772091.1
B4073	*Bacillus subtilis*	NZ_JXHP0.000037	WP_041338585.1	WP_142350264.1
SMRC103612	*Bacillus subtilis*	CP035406.1	WP_059293807.1	WP_139236146.1

b

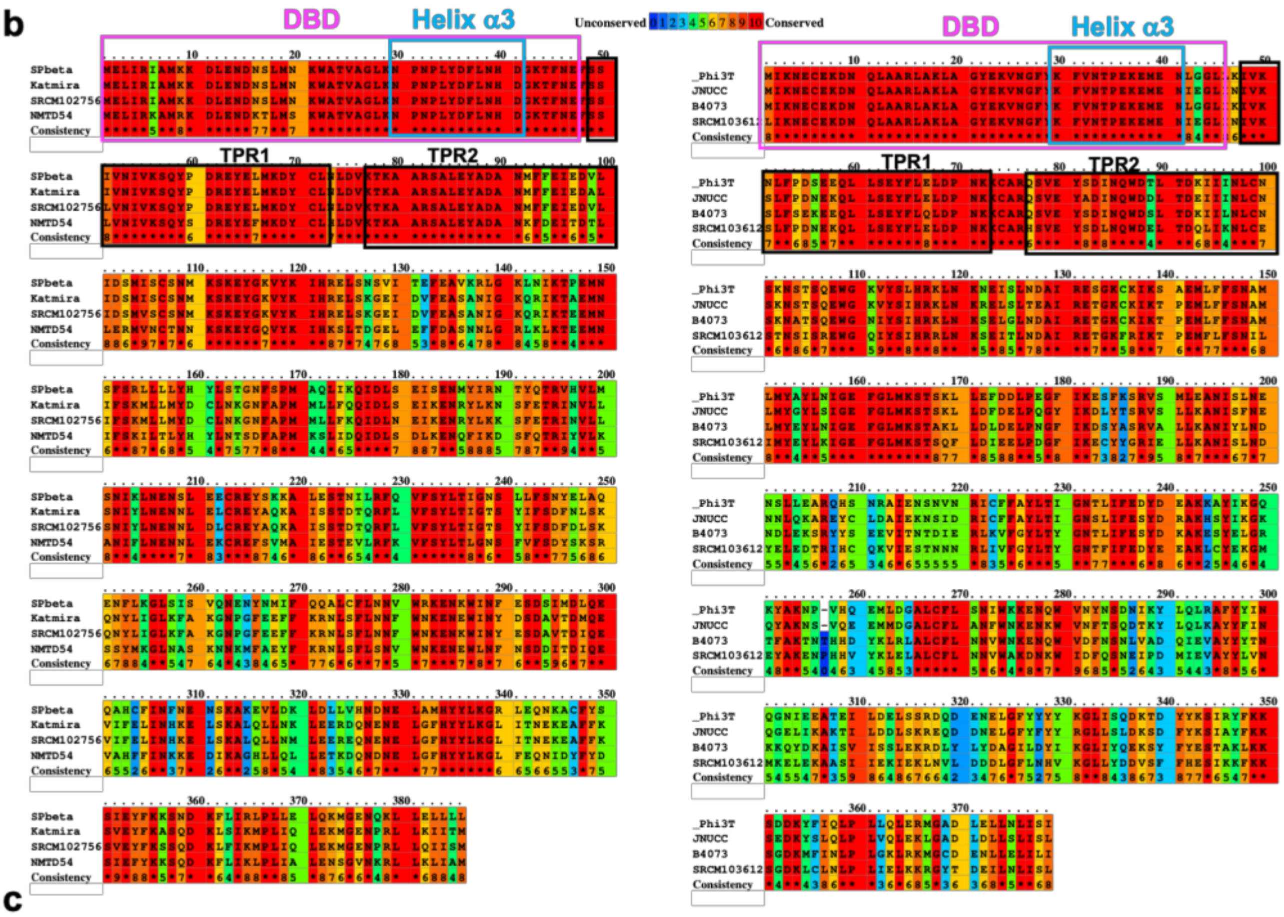

c

SPbeta	ATCACTTAAATATTAGGTTTTAATAACATCTAGTGAT	Phi3T	AAGTTCCAGAAATTCAAAAATCAAAAAATAAGAACAT
Katmira	ATCACTTAAATATTAAGTTTTTATAACATCTAGTGAT	JNUCC	AAGTTCCAGGAATTCTAAAATTGAAAAATGAGAACAT
SRCM102756	ATCACTTAAATATTAAGTTTTTATAACATCTAGTGAT	B4073	AAGTTCCAGAAATTCAAAAATCAAAAAATAAGAACAT
NMTD54	ATCACGAGATAAATTCAATTTCGCATAACTAAGGGAT	SMRC103612	AAGTTCCAGGAATTCTAAAACTGAAAAATGAGAACAT

d

SPbeta	MKKLIMALVILGALGTSYISAD-SSIQQASGDYEVAGMPRGA	Phi3T	MKKVFFGLVILTALAISFVAGQQSVSTASASDEVTVASAIRGA
Katmira	MKKFIMAITIAAVLSISFVGAKASSNEQASGDYQVAGIVRGA	JNUCC	MKKVIYGLMIIAALAVSFVAGQQSVSTASTSDEISVASIIRGA
SRCM102756	MKKFIMAIAIAAVLSISFVGAKASSNEQASGDYEVAGVVRGA	B4073	MKKVLYSLIIVIALAVGFVGGQKSMETASVDQPIKVASPSRGA
NMTD54	MKKVIMSALIVSAVALAFVGSNINKSNEASEEYNVAGMVRGA	SMRC103612	MKKIYFGLVILLALAVGFVSGQQSVETA--SGDVTVASASRGA

AimR receptors chimerity. a Arbitirum system compared showing similarities to those of SPβ (green background) or Phi3T (blue background). **b-d** Alignments of AimR receptors (**b**), DNA operators (**c**) and AimPs peptides (**c**) for systems showing chimerity trails with SPβ (*left*) and Phi3T (*right*). AimR alignments in **b** were performed with PRALINE (matrix BLOSUM62) server and are colored according to relative conservation. The location of DBD, helix α3, TPR1 and TPR2 are highlighted in boxes and labelled. Palindromic sequences for the DNA operators are highlighted in (**c**), as well as mature peptides (**d**)

Supplementary Tables

Supplementary Table 1. Data collection and refinement statistics

	$AimR^{Kat}$	$AimR\text{-}AimP^{Kat}$	$AimR^{Kat}$-DNA
**Data collection**			
Space group	$P2_12_12_1$	$C222_1$	$P2_1$
Cell dimensions			
a, *b*, *c* (Å)	77.58, 98.30, 144.35	69.70, 209.76, 140.25	132.72, 39.87, 143.01
α, β, γ (°)	90, 90, 90	90, 90, 90	90, 100.49, 90
Resolution (Å)	72.2-2.4 (2.46-2.4)*	144.1-2.6 (2.77-2.7)	65.3-2.5 (2.57-2.5)
R_{pym}	0.03 (0.40)	0.05 (0.61)	0.04 (0.73)
$I / \sigma I$	13.5 (1.9)	9.5 (1.2)	10.6 (1.0)
Completeness (%)	99.7 (100)	99.8 (99.9)	96.4 (75.8)
Redundancy	12.6 (13.5)	4.7 (5.0)	6.4 (5.3)
**Refinement**			
Resolution (Å)	2.4	2.6	2.5
No. reflections	43780(4329)	28616 (2830)	50316 (3867)
R_{work} / R_{free}	0.22(0.33)/ 0.25(0.37)	0.18 (0.31)/ 0.22(0.31)	0.20(0.40)/ 0.25(0.39)
No. atoms			
Macromolecules	6455	6434	8226
Ligand/ion	20	12	35
Water	67	11	49
B-factors			
Macromolecules	60.70	66.50	91.05
Ligand/ion	114.20	96.00	141.80
Water	65.80	67.30	70.90
R.m.s. deviations			
Bond lengths (Å)	0.014	0.014	0.016
Bond angles (°)	1.734	1.760	1.93

*Values in parentheses are for highest-resolution shell.

Supplementary Table 2. Strains and plasmids used in this study.

Strain or plasmid	Genotype	Reference
**Strains**		
B. subtilis strain 168	*trpC2*	[1]
B. subtilis Δ6	*trpC2; ΔSPβ; sublancin 168-sensitive; Δskin; ΔPBSX; Δprophage1; pks::Cm; Δprophage 3; Cmr*	[2]
B. subtilis subsp. KATMIRA 1933		[3]
BKK20860	trpC2 Δ*yop*K::kan s	[4]
JP19877	Δ6 lysogenic SPbeta	This work
JP19936	Δ6 Δ*aim*$R_{SP\beta}$	This work
JP19982	JP19877 *amyE*::P_{spank}	This work
JP20009	JP19877 *amyE*::P_{spank}-*aim*$R_{SP\beta}$	This work
JP19944	Δ6 *amyE*::P_{spank}-*aim*$R_{SP\beta}$	This work
JP20222	JP19936 *amyE*::P_{spank}	This work
JP20223	JP19936 *amyE*::P_{spank}-*aim*$R_{SP\beta}$	This work
JP20224	JP19936 *amyE*::P_{spank}-*aim*R_{Kat}	This work
JP20147	JP19936 *amyE*::P_{spank}-*aim*$R_{SP\beta\ 6xHis\ Ct}$	This work
**Plasmids**		
pDR244	*B. subtilis* temperature-sensitive plasmid with constitutively expressed Cre recombinase	[4]
pMiniMAD2	*B. subtilis* temperature-sensitive plasmid with erythromycin resistance	[5]
pDR110	*B. subtilis amyE* integration vector containing IPTG-inducible P_{spank} promoter	[6]
pJP2340	pDR100 *aim*$R_{SP\beta}$	This work
pJP2341	pDR100 *aim*R_{SPKat}	This work
pJP2342	pDR100 *aim*$R_{SP\beta\ 6xHis\ Ct}$	This work
pLIC-SGC1	pET expression vector with N-terminal His_6 and TEV protease cleavage site. Includes sites for LIC cloning.	Addgene plasmid # 39187
pLIC-AimRKat33	pLIC containing *aim*R^{kat} cloned	This work
pLIC-AimRKat33^{N273A}	pLIC containing *aim*R^{kat} with mutation N273A cloned	This work
pLIC-Aim$R^{SP\beta}$	pLIC containing *aim*$R^{SP\beta}$ cloned	[7]
PET21b	pET expression vector	Novagene
pET-Aim$R^{SP\beta}$-II	pET21b containing *aim*$R^{SP\beta}$ cloned	This work

Supplementary Table 3. Oligonucleotides used in this study.

Oligo	Sequence
**Cloning in pDR110 plasmid**	
AimR_pDR110_FW	CCCAAGCTTGACTCGTAATGTGATCTATAG
$AimR^{SP\beta}$_pDR110_RV	ACGCGTCGACCATTGTCTCACCTCCTTTAAAGTAAAAG
$AimR^{Kat}$_pDR110_RV	ACGCGTCGACCACCTCCTTTCATTATTAAGTTTACATAG
$AimR^{SP\beta}$ 6xHis - pDR110_RV	ACGCGTCGACCATTGTCTCACCTCCTTTAGTGGTGGTGGTGGTGGTGTTCAAGAAGTAAAAGTAATTCTAAAAGTTTTTGATTTTCACCC
**Cloning in pLicSGC1 plasmid**	
Kat33Plic_FW	TACTTCCAATCCATGGAGTTAATAAGGATAGCTATGAAGAAAG
Kat33Plic_RV	TATCCACCTTTACTGTTACATAGTAATAATCTTTAGTAATCTTGGATTTTC
**Cloning in pET21b plasmid**	
SPbCter_FW	CAGTGGTGGTGGTGGTGGTGCTCAAGTAAAAGTAATTCTAAAAGTTTTTG
SPbCter_RV	GAGATATACATATGGCTAGCATGGAGTTAATAAGGATAGC
pet21_FW	CACCACCACCACCACCAC
pet21_RV	GCTAGCCATATGTATATCTCCTTCTTAAAGTTAAAC
**AimR mutants**	
$Kat33^{N273A}$_FW	TTTAAGTTTTTTAAACAATTTTTGGAAC
$Kat33^{N273A}$_RV	GCTCTTTGAAAAACTCCTCAAACC
**Double stranded DNA probes for EMSA**	
DNA_kat	GTTGATCACTTAAATATTAAGTTTTTATAACATCTAGTGATGGCC
DNA_SPβ	GTTGATCACTTAAATATTAGGTTTTAATAACATCTAGTGATGGCC
DNA_Phi3T	GTTGATGTTCCAGAAATTCAAAAATCAAAAAATAAGAACATGGCC
**Biotinylated double stranded probes for Biolayer Interferometry**	
DNA_SPβ_b	GTTGATCACTTAAATATTAGGTTTTAATAACATCTAGTGATGGCC
DNA_Phi3T_b	GGGAAAGTTCCAGAAATTCAAAAATCAAAAAATAAGAACATGGGG
DNA_Kat_b	GTTGATCACTTAAATATTAAGTTTTTATAACATCTAGTGATGGCC

References

1. Zeigler, D. R. *et al.* The origins of 168, W23, and other Bacillus subtilis legacy strains. *Journal of bacteriology* **190**, 6983–95 (2008).

2. Westers, H. *et al.* Genome Engineering Reveals Large Dispensable Regions in Bacillus subtilis. *Molecular Biology and Evolution* **20**, 2076–2090 (2003).

3. Karlyshev, A. v., Melnikov, V. G. & Chikindas, M. L. Draft genome sequence of Bacillus subtilis strain KATMIRA1933. *Genome Announcements* **2**, 619–633 (2014).

4. Koo, B.-M. *et al.* Construction and Analysis of Two Genome-Scale Deletion Libraries for Bacillus subtilis. *Cell Systems* **4**, 291-305.e7 (2017).

5. Patrick, J. E. & Kearns, D. B. MinJ (YvjD) is a topological determinant of cell division in *Bacillus subtilis*. *Molecular Microbiology* **70**, 1166–1179 (2008).

6. Carniol, K., Ben-Yehuda, S., King, N. & Losick, R. Genetic dissection of the sporulation protein SpoIIE and its role in asymmetric division in Bacillus subtilis. *Journal of bacteriology* **187**, 3511–20 (2005).

7. Gallego del Sol, F., Penadés, J. R. & Marina, A. Deciphering the Molecular Mechanism Underpinning Phage Arbitrium Communication Systems. *Molecular Cell* **74**, 59-72.e3 (2019).
